# Long-Term Temporal Analysis of the Human Fecal Microbiota Revealed a Stable Core of Dominant Bacterial Species

**DOI:** 10.1371/journal.pone.0069621

**Published:** 2013-07-16

**Authors:** Inés Martínez, Catherine E. Muller, Jens Walter

**Affiliations:** Department of Food Science and Technology, University of Nebraska, Lincoln, Nebraska, United States of America; Argonne National Laboratory, United States of America

## Abstract

Next-generation sequencing has greatly contributed to an improved ecological understanding of the human gut microbiota. Nevertheless, questions remain regarding the characteristics of this ecosystem and the ecological processes that shape it, and controversy has arisen regarding the stability of the bacterial populations and the existence of a temporal core. In this study, we have characterized the fecal microbial communities of three human individuals over a one-year period by 454 pyrosequencing of 16S rRNA tags in order to investigate the temporal characteristics of the bacterial communities. The findings revealed a temporal core of 33 to 40 species-level Operational Taxonomic Units (OTUs) within subjects. Although these OTUs accounted only for around 12% of the total OTUs detected, they added up to >75% of the total sequences obtained for each individual. In order to determine the capacity of the sequencing and bioinformatic approaches applied during this study to accurately determine the proportion of a core microbiota, we analyzed the fecal microbiota of nine mice with a defined three-member community. This experiment revealed that the sequencing approach inflated the amount of rare OTUs, which introduced a significant degree of artificial variation across samples, and hence reduced the apparent fraction of shared OTUs. However, when assessing the data quantitatively by focusing on dominant lineages, the sequencing approaches deliver an accurate representation of the community. In conclusion, this study revealed that the human fecal microbiota is dominated by around 40 species that maintain persistent populations over the duration of one year. The findings allow conclusions about the ecological factors that shape the community and support the concept of a homeostatic ecosystem controlled largely by deterministic processes. Our analysis of a three-member community revealed that methodological artifacts of OTU-based approaches complicate core calculations, and these limitations have to be considered in the interpretation of microbiome studies.

## Introduction

The gastrointestinal tract of humans is colonized by a complex microbial community dominated by bacteria referred to as the gut microbiota. The bacterial cells in the gastrointestinal tract outnumber somatic cells by at least an order of magnitude, so it is not surprising that the gut microbiota is of profound importance for human health and physiology [1]. Alterations of the gut microbiota have been linked to several chronic immunological and metabolic diseases in humans (including obesity, heart disease, colon cancer, and a variety of inflammatory conditions), and in several animal models, aberrations in gut microbiota composition play a causative role in the development of these pathologies [2], [3], [4], [5]. The association of the gut microbiota with human disease opens avenues for the development of therapies that aim to restore the ecosystem, but their implementation requires a mechanistic understanding about the ecological principles that shape and regulate microbial communities [6].

It is becoming increasingly clear that our understanding of the human microbiome will require the application of ecological theory, and the development of concepts that apply specifically to host associated microbial communities [7], [8]. To be successful, this will first require a thorough characterization of the communities in terms of temporal and spatial diversity in different environmental contexts. Despite several decades of research, conflicting perspectives remain regarding the nature of the human gut microbiota, with older concepts being challenged in light of new evidence [9]. In a classic review, Dwayne Savage considered the assembly of the gut microbiota a predictable niche-driven process that ultimately results in the establishment of a climax community with a high degree of temporal stability [10]. In his model, which is essentially the classic deterministic ecological perspective [11], niches characterized by nutrients, environmental filters, and the principle of competitive exclusion determine species membership, abundance, distributions and diversity, and are ultimately stably occupied by the best adapted competitor. Savage referred to these community members as ‘autochthonous’, and they were not only expected to maintain stable populations in normal adults but were also supposed to be always detected in individuals of the host species [12]. This classic deterministic view has undeniable success in providing explanations for some ecological characteristics of gut microbiomes, such as the presence of specific communities with particular traits in different intestinal compartments and the occurrence of colonization resistance [13], [14]. In addition, some aspects of Savage’s early concepts were more recently supported by findings indicating that individual members can be detected in a majority of individuals [15], [16], [17], that mammals assemble gut microbial communities whose composition is phylogenetically conserved [18], that gut microbes can show specific adaptations to the niche environment in particular hosts [19], and that whole communities show a remarkable stability [20], [21], [22], [23], [24].

However, new concepts in community ecology and findings obtained through next-generation sequencing techniques have challenged the conventional concepts of host-associated microbial communities [9]. The profiling of ribosomal RNA (rRNA) sequences, which exceeds previous techniques in terms of phylogenetic resolution and dynamic range, has suggested that the human fecal microbiota is highly individualized at the species level, even among monozygotic twins [25]. The factors driving the substantial inter-subject variation observed in humans have not been determined yet, but modern concepts of community ecology presume that, apart from deterministic factors, historic and neutral processes can contribute significantly to ß-diversity [11]. The debate as to what extent gut microbiomes are shaped by deterministic, neutral, and historic processes falls within the larger historic controversy about the nature of communities, and an assessment of their relative importance is essential for our ability to explain and predict community structure and dynamics and ultimately develop models and ecological theories. In this respect, it is important to point out that communities shaped largely by either historic or neutral principles would have fundamentally different characteristics. A community whose assembly is largely impacted by historic processes would be niche-centered, although niches would not be determined solely by environmental filters but also physicochemical changes of the habitat caused by community members, species interactions, and adaptive processes during assembly [26]. The niche-environment would be influenced by the historic trajectory of the assembly process, which is inherently stochastic and could account for ß-diversity [14]. Despite the importance of non-deterministic factors, specialization for habitats would still play a central role in determining diversity and species abundance, and in the assembled community, niches would be stably occupied by what is the best adapted organism. In contrast, the neutral model presumes that communities are composed of species that are ecologically equivalent, and composition at a local scale and ß-diversity arise solely by dispersal limitation, disturbance, and other random processes [27]. Consequently, communities are open to additional colonists, are continuously changing, and have non-equilibrium assemblages of species which show functional redundancies [11]. Given the different characteristics of communities shaped by deterministic and neutral factors, temporal patterns of populations can be interpreted to deduce the ecological principles by which they are governed.

Recently, Caporaso and co-workers have provided the first long-term (two subjects, daily samples for 15 months) analysis of the bacterial communities at different body sites (feces, mouth, skin) employing Illumina sequencing and 454 pyrosequencing [28]. This work exceeded previous studies in sequencing depth, duration, and taxonomic resolution, and it revealed a pronounced temporal variability in an individual's fecal microbiota. Only a minor fraction of bacterial taxa were detected to be persistent, and the authors concluded that there is little evidence for a core temporal fecal microbiome. The findings of this study contradicted previous studies that were more short-term and relied on non-sequencing based methods with lower taxonomic resolution [20], [21], [22], [23], [24], [29], and suggested that the human gut microbiome might be significantly less stable than previously acknowledged. To gain an independent perspective on the long-term temporal dynamics of the human gut microbiome, we characterized the fecal bacterial communities of three human individuals over the duration of one year to the species level. This was achieved by analyzing sequence data sets generated with 454 pyrosequencing of 16S rRNA tags during two consecutive nutritional studies [30], [31]. In agreement with Caporaso and colleagues, we found only a small proportion of species-level OTUs that persisted over a one-year period. However, by employing a quantitative approach, we arrived at different conclusions in terms of the long-term temporal characteristics of the human microbiota, and a temporal core that dominated the fecal microbiota was identified. To evaluate the ability of the OTU-selection approach used in this study to accurately determine the proportion of a core microbiota, we characterized the fecal microbiota of nine mice with a defined three-member community. This experiment revealed that the sequencing approach inflated diversity measures and reduced the apparent fraction of shared OTUs, and these methodological artifacts were considered in the interpretation of the human data.

## Results

### General Characteristics of the Fecal Microbiota in Three Human Individuals

**Figure 1:**
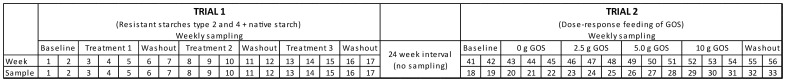
Experimental design. 33 (two subjects) and 32 (one subject) samples were collected in a 56-week time period from human subjects that participated in two dietary trials testing the effect of resistant starches and GOS on the fecal microbiota [31], [32]. Fecal samples were collected weekly throughout both trials (17 in Trial 1 and 16 in trial 2), and the two trials were interspaced by a 24-week period without sampling.

To investigate the temporal patterns of the human fecal microbiota ove[r a 56-week period, we characterized the bacterial populations in three healthy subjects that participated in two dietary trials that tested the impact of resistant starches (types 2 and 4) and GOS on the bacterial populations [31], [32]. The sequencing approach used resulted in sequences of 500 bp spanning the entire V1-V3 region of the 16S rRNA gene, allowing clear assignments to the species level in most cases. 33 fecal samples from subjects 1 and 2, and 32 samples from subject 3, were analyzed (see Figure 1 for an overview of the study design). All three subjects remained healthy throughout the entire study period with the exception of minor infections. Subject 1 underwent a 1-week antibiotic treatment during the 24-week period in which no sampling took place (week 19, corresponding to the 2^nd^ week of the non-sampling period). Subject 3 did a deliberate lifestyle change between the dietary trials that included an increase in exercise, healthier nutrition, and moderate weight loss (7 kg).

As shown in many previous studies, the subjects’ microbiota were conserved at the phylum level and dominated by Firmicutes and Bacteroidetes phyla, and to a lesser degree Actinobacteria, Verrucomicrobia and Proteobacteria. At lower taxonomic scales, the microbiota was individualized and clustered by subject (Figure S1). The lifestyle changes adopted by subject 3 were reflected in the separate clustering of the fecal microbial community among samples taken in the two trial periods (but were still distinct from those of the other subjects), and was associated with a pronounced decrease in the Firmicutes:Bacteroidetes ratio (from 20±10 in the first study period, to 2±1 in the second trial). Equivalent changes of this ratio have been linked to weight loss in a previous study [33], but the mechanisms that cause these shifts are not yet understood.

### Temporal Dynamics of the Human Fecal Microbiota at Different Taxonomic Scales

Sequence proportions determined by pyrosequencing were used to characterize the temporal dynamics of the fecal microbiota within individuals. In accordance with previous studies [24], [28], the temporal stability of the fecal microbiota is dependent on the taxonomic scale, decreasing from the phylum to the species level (Figure S2). To determine the temporal core at the species level, we identified the OTUs that were detected in at least 80% of the samples within an individual. The criteria for identifying core members are not standardized, and the criteria used in our study differed from that used by Caporaso and colleagues [28], who defined persistent core members as those that were observed across all sampling events, ignoring single, isolated zero-counts. However, this standard might be too stringent as it would select against core members that become temporarily undetectable when dropping below the detection threshold (which accounts for around 10^7^ cells/gram with the sequencing depth obtained in our study). Given the dynamic nature of the gut microbiota and the limited analysis depth, we considered OTU detected in >80% of the samples as members of the temporal core.

**Figure 2:**
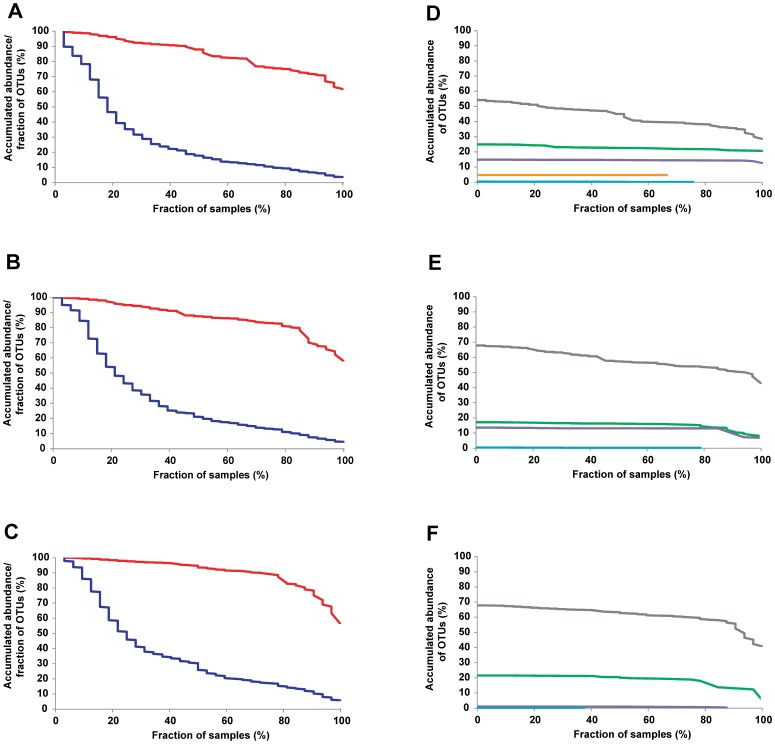
Individual fecal temporal core. Fraction of species-level OTUs shared across samples of the total OTUs within these samples (blue line) and the proportion that these OTUs represent of the total sequences obtained (red line) for subjects 1, 2, and 3 (A,B,C). Proportion of total sequence reads obtained of OTUs shared across sampled within the different phyla (Firmicutes: grey, Bacteroidetes: green, Actinobacteria: purple, Verrucomicrobia: orange, Proteobacteria: light blue) (D,E,F).

Only a small proportion of the total species-level OTUs were determined to make up an individual’s temporal core (Figure 2). Out of the total of 411±119 OTUs detected per subject, 12% ±3% of the OTUs were detected as stable. Overall, 69 OTUs were determined to be persistent in at least one subject (39, 40 and 33 in the individual subjects), out of which 16 were shared among the 3 subjects (Table 1). It is important to point out that the majority of these OTUs were detected throughout the entire period. Only 6 OTUs became undetectable in 3 consecutive weeks (1 in subject 1, 2 in subject 2, and 3 in subject 3) and only one OTU in subject 2 was undetected in 4 consecutive weeks.

**Table 1:** Abundance (%) of bacterial OTUs that comprise the temporal core within the fecal microbiota of three humans over a one-year period.

OTU #	Phylum	Closest related type strain*(average percent similarity)	Subject 1 Mean ± SD (percent of samples in which OTU was detected)	Subject 2 Mean ± SD (percent of samples in which OTU was detected)	Subject 3 Mean ± SD (percent of samples in which OTU was detected)
2	Actinobacteria	*Bifidobacterium adolescentis* (100)	8.67±7.15 (100)	5.80±11.47 (88)	ND
5	Actinobacteria	*Bifidobacterium adolescentis* (96)	3.91±3.32 (100)	0.04±0.08 (27)	ND
358	Actinobacteria	*Bifidobacterium pseudocatenulatum* (99)	0.13±0.26 (42)	3.68±2.89 (100)	ND
11	Actinobacteria	*Bifidobacterium longum* (99)	1.19±0.92 (97)	0.44±0.47 (94)	0.28±0.42 (84)
928	Actinobacteria	*Adlercreutzia equilifaciens* (98)	ND	ND	0.39±0.51 (91)
40	Actinobacteria	*Collinsella aerofaciens* (98)	0.50±0.43 (97)	3.15±5.20 (100)	0.34±0.57 (47)
16/548	Bacteroidetes	*Bacteroides thetaiotaomicrom* (98)	1.78±0.81 (100)	0.40±0.31 (91)	0.09±0.14 (50)
20	Bacteroidetes	*Bacteroides xylanisolvens* (99)	4.39±4.49 (100)	0.32±0.38 (79)	ND
273	Bacteroidetes	*Bacteroides intestinalis* (98)	ND	0.81±0.61 (97)	ND
18	Bacteroidetes	*Bacteroides uniformis* (99)	3.37±1.78 (100)	1.29±1.14 (100)	0.57±0.70 (66)
1	Bacteroidetes	*Bacteroides vulgatus* (99)	8.98±5.09 (100)	5.15±3.98 (100)	7.11±8.24 (97)
512	Bacteroidetes	*Bacteroides dorei* (100)	0.10±0.22 (21)	0.56±0.38 (97)	0.80±1.19 (75)
269	Bacteroidetes	*Bacteroides ovatus* (97)	0.15±0.20 (67)	0.25±0.33 (82)	0.06±0.09 (50)
118	Bacteroidetes	*Odoribacter splanchnicus* (99)	0.17±0.14 (88)	0.36±0.33 (82)	0.08±0.15 (44)
226	Bacteroidetes	*Parabacteroides distasonis* (97)	0.35±0.61 (85)	0.14±0.18 (70)	1.43±2.88 (85)
513	Bacteroidetes	*Alistipes shahii* (100)	0.03±0.07 (21)	0.22±0.18 (91)	0.14±0.18 (53)
235	Bacteroidetes	*Alistipes finegoldii* (100)	0.42±0.49 (88)	ND	0.07±0.10 (50)
19	Bacteroidetes	*Bacteroides putredinis* (99)	2.14±1.16 (100)	1.62±1.33 (100)	5.29±3.80 (100)
479	Bacteroidetes	*Bacteroides intestinihominis* (99)	0.14±0.30 (24)	0.56±0.47 (97)	ND
4400/4617	Bacteroidetes	Bacteroidales	ND	2.83±2.92 (91)	ND
114	Firmicutes	*Flavonifactor*	0.11±0.13 (70)	0.04±0.05 (52)	0.40±0.35 (91)
12556	Firmicutes	Ruminococcaceae bacterium D16 (97)**	ND	ND	0.44±0.39 (91)
359	Firmicutes	*Oscillibacter* spp.	0.09±0.09 (67)	0.16±0.15 (82)	0.15±0.16 (66)
5715	Firmicutes	Ruminococcaceae	0.02±0.04 (15)	0.03±0.07 (27)	0.50±0.54 (94)
32	Firmicutes	*Butyricicoccus* spp.	0.21±0.19 (91)	0.03±0.06 (39)	ND
2817	Firmicutes	Clostridiales	ND	0.55±0.74 (85)	ND
5522	Firmicutes	*Ruminococcus bromii* (99)	0.80±1.92 (21)	1.02±3.95 (18)	4.90±5.32 (100)
4705	Firmicutes	Ruminococcaceae	0.09±0.25 (21)	0.41±0.46 (85)	0.16±0.28 (50)
4256	Firmicutes	Ruminococcaceae	1.48±3.07 (45)	4.56±3.73 (100)	3.74±3.29 (100)
161/4277	Firmicutes	Clostridiales	0.12±0.12 (85)	0.22±0.18 (88)	ND
2308	Firmicutes	Clostridiales	0.14±0.24 (58)	1.33±0.90 (94)	ND
29	Firmicutes	*Faecalibacterium prausnitzii* SL3/3 (99)**	1.36±1.17 (100)	2.01±1.30 (100)	3.05±2.22 (100)
35	Firmicutes	*Faecalibacterium prausnitzii* KLE-1255 (98)**	0.85±0.77 (97)	0.03±0.04 (45)	0.03±0.07 (22)
6	Firmicutes	*Faecalibacterium prausnitzii* KLE-1255 (97)**	2.72±2.20 (100)	1.51±1.46 (100)	1.60±1.33 (97)
17	Firmicutes	*Faecalibacterium prausnitzii* A2-165 (97)**	4.68±2.23 (100)	3.56±1.67 (100)	2.65±3.10 (100)
31/136	Firmicutes	*Dialister* spp.	0.41±0.29 (91)	ND	ND
288	Firmicutes	*Dialister invisus* (100)	ND	ND	3.07±2.68 (91)
66	Firmicutes	*Streptococcus salivarius* (98)	0.73±0.66 (94)	0.27±0.45 (64)	0.64±0.87 (94)
21	Firmicutes	*Streptococcus parasanguinis* (99)	0.22±0.36 (88)	0.02±0.05 (24)	0.04±0.07 (34)
22	Firmicutes	*Clostridium spiroforme* (99)	0.18±0.20 (73)	ND	0.17±0.20 (81)
53	Firmicutes	Erysipelotrichaceae	0.43±0.33 (88)	ND	ND
9864	Firmicutes	Erysipelotrichaceae	ND	2.45±1.97 (100)	1.21±1.35 (100)
9902	Firmicutes	Erysipelotrichaceae	ND	0.14±0.11 (81)	ND
48/23/365/27/262/10	Firmicutes	*Ruminococcus obeum* (98)	3.13±1.56 (100)	7.46±3.74 (100)	5.73±3.53 (100)
3/7	Firmicutes	*Blautia wexlerae* (99)	6.62±5.24 (100)	1.87±1.95 (100)	6.02±4.24 (100)
361	Firmicutes	Lachnospiraceae	0.12±0.12 (82)	0.11±0.11 (70)	0.12±0.15 (69)
9873	Firmicutes	*Dorea formicigenerans* (97)	ND	0.05±0.07 (48)	0.42±0.49 (97)
51	Firmicutes	*Dorea formicigenerans* (99)	0.79±0.43 (100)	0.62±0.49 (100)	2.62±3.07 (100)
76/4328	Firmicutes	*Dorea* spp.	0.23±0.21 (85)	0.51±0.25 (100)	1.04±0.81 (100)
78	Firmicutes	*Ruminococcus torques* (97)	0.04±0.13 (27)	0.04±0.13 (27)	0.89±1.20 (91)
9942	Firmicutes	*Ruminococcus torques* (99)	ND	0.19±0.17 (88)	0.08±0.13 (50)
24/511/403	Firmicutes	*Coprococcus comes* (98)	0.57±0.51 (97)	3.01±2.01 (100)	3.86±1.99 (100)
12	Firmicutes	*Ruminococcus gnavus* (99)	0.36±0.29 (94)	0.07±0.19 (36)	0.14±0.15 (72)
26	Firmicutes	*Eubacterium ramulus* (99)	0.64±0.34 (97)	0.28±0.29 (91)	0.82±1.04 (91)
42	Firmicutes	*Roseburia inulinivorans* (100)	0.26±0.27 (91)	0.17±0.37 (55)	1.44±1.55 (91)
120	Firmicutes	Lachnospiraceae	0.36±0.25 (97)	0.04±0.05 (52)	0.09±0.11 (53)
14/1107	Firmicutes	Lachnospiraceae	2.53±1.64 (100)	4.61±2.32 (100)	1.38±1.15 (94)
63/370	Firmicutes	*Clostridium clostridiiformes* (96)	1.24±1.58 (100)	4.42±3.18 (100)	2.34±2.70 (100)
9865	Firmicutes	*Coprococcus euctatus* (99)	ND	3.79±2.91 (100)	ND
3927	Firmicutes	*Coprococcus catus* GD/7 (98)**	0.08±0.10 (48)	0.15±0.13 (91)	ND
30	Firmicutes	Lachnospiraceae	0.21±0.27 (82)	0.03±0.11 (18)	0.01±0.03 (25)
9	Firmicutes	Lachnospiraceae	0.83±0.93 (82)	0.04±0.08 (36)	0.13±0.15 (56)
4264	Firmicutes	*Eubacterium eligens* (100)	0.06±0.16 (41)	0.30±0.25 (91)	0.12±0.11 (63)
4	Firmicutes	*Eubacterium rectale* (97)	6.49±5.44 (100)	9.07±9.32 (100)	4.06±3.53 (100)
4262/2326	Firmicutes	*Roseburia intestinalis* (98)	2.26±2.72 (55)	0.56±1.36 (27)	4.26±3.40 (100)
45	Firmicutes	Lachnospiraceae	1.03±0.67 (100)	0.29±0.36 (70)	0.19±0.37 (69)
15	Firmicutes	*Clostridium* sp. SS2/1 (98)**	1.50±1.19 (100)	0.24±0.26 (85)	0.80±1.09 (100)
5348	Firmicutes	*Eubacterium ventriosum* (97)	0.06±0.16 (18)	0.26±0.22 (79)	0.41±0.48 (88)
36	Verrucomi crobia	*Akkermansia muciniphila* (98)	4.70±7.15 (67)	0.30±0.94 (36)	5.82±8.92 (100)
		**Sum**	**85.17%**	**84.44%**	**82.19%**

Mean and standard deviations were calculated with all samples across the study period.

* Similarity was assessed by alignment of the sequences with ClustalW (p-distances).

** Not the type strain.

### The Human Fecal Microbiota is Dominated by a Small Number of Persistent Species

The small proportion of OTUs that were stably detected over a 56-week time period could give the impression of a negligible temporal core within the human fecal microbiota. However, when the abundance of these OTUs is considered (calculated from the proportion of sequences represented by each OTU), the analysis revealed that the majority of the human fecal microbiota is stably maintained (Figures 2), as 75%, 81%, and 79% of the total sequences from the individual subjects belonged to stable OTUs (Figure 2A to C). Moreover, even if only OTUs detected in 100% of the samples within a subject are considered (18, 19, and 16 OTUs for subjects 1, 2, and 3, respectively), they composed 62% ±4% of the total microbiota, on average. In addition, the 16 stable OTUs that were shared among the three subjects comprised an average of 47% of the total reads per individual. Thus, the proportion of the inter-individual core microbiota in our study is comparable to that identified in a previous human study [17].

The 69 species that remained stable in at least one of the subjects (Table 1) belonged to the four phyla Firmicutes (48 OTUs), Bacteroidetes (14 OTUs), Actinobacteria (6 OTUs), and Verrumicrobia (1 OTU). Out of the 16 stable OTUs that were detected in all 3 subjects, twelve showed >97% sequence similarity to described species: *Bifidobacterium longum*, *Bacteroides vulgatus*, *Bacteroides putredinis*, *Faecalibacterium prausnitzii* (3 OTUs, related to strains SL3/3, KLE1255, and A2-165), *Ruminococcus obeum*, *Blautia wexlerae*, *Dorea formicigenerans*, *Eubacterium ramulus*, *Eubacterium rectale*, and *Coprococcus comes*. Another 11 OTUs were stably detected in 2 of the 3 subjects, and included phylotypes closely related to *Bifidobacterium adolescentis*, *Collinsella aerofaciens*, *Bacteroides thetaiotaomicron*, *Bacteroides uniformis*, *Odoribacter splanchnicus*, *Parabacteroides distansonis*, *Streptococcus salivarius*, *Roseburia inulinivorans*, as well as one additional lineage within each the Ruminococcaceae, Clostridiales and Erysipelotrichaceae (Table 1). Out of these 11 OTUs, 7 were also detected in the third subject, but in less than 80% of the samples. Finally, 42 OTUs were determined to be persistent only in one individual, although 32 of these OTUs were transiently detected in at least one other subject (Table 1). Firmicutes comprised most of the transient members of the fecal microbiota, whereas Actinobacteria, Bacteroidetes and Verrucomicrobia populations remained more stably associated to the gut ecosystem (Figure 2D to F).

### Resilience of Core Members to Short-term Dietary Modulations

Diet is a major factor in shaping the structure of the gut microbiota [34], and long-term dietary preferences have been linked to consistent differences in the microbial community structure among humans [35]. As previously described, the dietary modulations used in the two trials (RS2, RS4, native starch, and GOS) had individualized and reversible effects on the fecal microbiota of the subjects [30], [31], [32]. Interestingly, the most substantial diet-driven changes detected concerned species of the temporal core, namely *Bifidobacterium adolescentis* (induced through RS2, RS4, and GOS) and *Parabacteroides distasonis* (induced through RS4). As shown in Figure S3, the diet-induced changes in the abundance of these taxa was individualized, and tightly linked to the dietary modulation, with populations returning to baseline levels after treatment cessation. These findings provide evidence that shifts within core members can be induced though diet but populations show resilience.

### Characterization of the Temporal Fluctuations in Relative Abundance of Core Members

**Figure 3:**
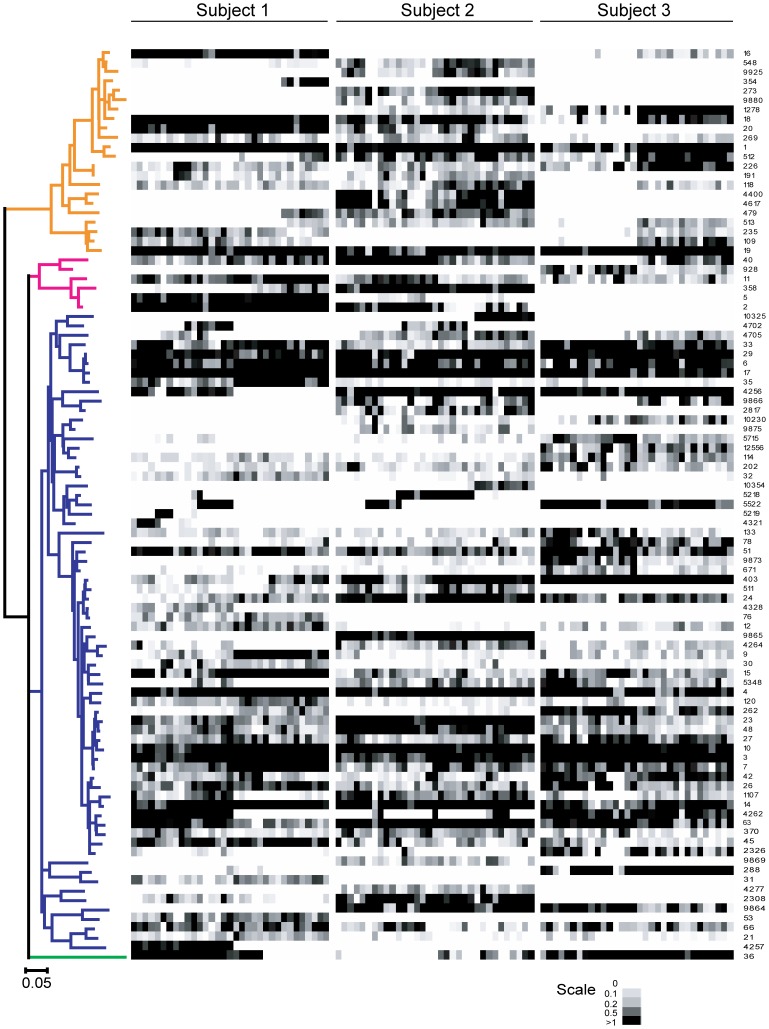
Abundance of the dominant bacterial OTUs in the fecal samples of subjects throughout the study. All OTUs that had an average proportion >0.2% were included. Representative sequences of OTUs were used to construct the Maximum Likelihood phylogenetic tree, which is colored-coded according to phylum: Bacteroidetes (orange), Actinobacteria (magenta), Firmicutes (blue), Verrucomicrobia (green). The abundance of each phylotype is indicated by grayscale. The OTU numbers are presented to the right of the heatmap.

Virtually all members of the human fecal microbiota, including members of the temporal core, showed temporal fluctuations in relative abundance (Figure 3 and S4–S7). Normal variations in the individual’s diet are one likely cause of these patterns. Accordingly, the taxa with the highest fluctuations belong to species that have been identified to respond to dietary compounds or possess enzymatic capabilities to degrade dietary carbohydrates. Examples are *Bacteroides xylanisolvens* (utilizes xylan), *Blautia wexlerae* (enriched by whole grains [36]), *Clostridium clostridiiformes* (enriched by RS4 [31]), *Eubacterium rectale* and *Ruminococcus bromii* (enriched by RS2 and RS3 [31], [37]), *Faecalibacterium prausnitzii* (enriched through the prebiotics inulin, oligofructose, GOS [34]), and several *Roseburia* species (enriched by whole grains [36]).

### Evidence for Events of Invasion and Extinction

Although the findings obtained during this study suggest that most of the dominant bacterial species in the human gut are persistent over a one-year time span, the analysis revealed several patterns that are indicative of invasion and extinction events. Such events were indicated by stable populations of OTUs over longer timespans that abruptly appeared or disappeared. For example, such patterns suggested the extinction of OTU 36 (*Akkermansia muciniphila*) in subject 1, and the invasion of the same species in subject 2 (Figures 3 and S8). In subject 1, several phylotypes among the Firmicutes (OTUs 4262, 4257, 4256) became extinct between the two nutritional trials (Figures 3 and S8). This “mass extinction” event coincided with a short-term clarithromycin treatment. Interestingly, all four OTUs are closely related to members of the fecal microbiota (*Clostridium clostidiiforme*, *Eubacterium ramulus*, *Eubacterium rectale*, *Eubacterium ventriosum* and *Roseburia cecicola)* that have been shown to be affected by long-term clarithromycin therapy [38].

Some population dynamics are suggestive of niche-related processes, potentially associated with cooperation or competition. For example, several distinct OTUs belonging to the Bacteroidetes emerge simultaneously after week 49 in subject 1 (OTUs 354, 479 and 513) (Figure 3), probably associated with the availability of a new niche or a syntrophic relationship between these species. In addition, within the Ruminococcaceae family, a direct succession of closely related OTUs was observed in subject 1 (OTU 4321 followed by 5219 and then 5522) and subject 2 (OTU 5522 followed by 5218 followed by 10354), suggesting events of invasion coupled with competitive exclusion of closely related taxa (Figure 3, Figure S8).

### Pyrosequencing Overestimates Diversity in a Defined Three-member Community, Artificially Augmenting β-diversity

As described previously [28], this study revealed that most OTUs detected by sequencing in human fecal samples are not stably detected over time. These OTUs could constitute species that are transient. However, limitations of sequence surveys, such as sequencing errors, chimeras, and over-splitting of OTUs, can result in artifacts that can confound the interpretation of community data, especially in terms of diversity determination [39], [40], [41] and the definition of a core [42]. To assess the capacity of the OTU-based sequencing approach used in our study to investigate measures of bacterial community diversity, we characterized the fecal microbiota in nine gnotobiotic mice with a simple defined microbiota composed of *Bacteroides thetaiotaomicron*, *Bifidobacterium adolescentis*, and *Escherichia coli*. Taxonomic classification of the sequences down to the genus level with the Classifier (RDP) tool identified 3 genera as being the only members of the fecal microbiota in the animals (Figure S9), with 19.7% of the sequences accounting for the genus *Bifidobacterium*, 20.3% for *Escherichia*, and 58.7% for *Bacteroides*. This showed that the Classifier algorithm (which performs taxonomic assignments of individual reads and is not reliant on multiple sequence alignments) is able to correctly assign most sequences to the bacterial genera that were present in these mice.

However, the OTU picking led to a substantial overestimation of diversity, as 237 OTUs were detected in the data set (an average of 42 per animal). Only 9 OTUs were detected in all 9 animals (Figure S9), which accounts for only 3.8% of the total OTUs. Therefore, the approach led to artificial measures of both α- and β-diversity, giving the false impression that the microbiota across these animals is highly variable and that the fraction of core species is very small. However, it is important to point out that the 9 OTUs detected in all samples represented virtually the entire bacterial population (96.6% of the sequences) (Figure S9). Importantly, the three most abundant OTUs accounted for the three individual species present in the mice, and comprised 79% of the total sequences, 17% for *Bifidobacterium adolescentis*, 17% for *Escherichia coli*, and 45% for *Bacteroides thetaiotaomicron*. In conclusion, this validation experiment showed that OTU picking in combination with sequencing errors overestimates the number of OTUs and β-diversity among samples.

## Discussion

The diversity and temporal dynamics are important characteristics of a bacterial community are a reflection of the ecological processes that shape the ecosystem. The human gut microbiota has historically been considered stable [10], [23], [29], but recent work using next-generation sequencing of 16S rRNA tags has challenged older findings and suggested a pronounced intra- and inter-subject variability [25], [28]. The new insight perpetuated the notion that the human gut microbiota is composed of hundreds of species that show little conservation, not only across individuals but also over time [6]. In a recent review, Fierer and coworkers argued that “it is apparent that the adult microbiome is in a constant state of flux as microbial community composition on and in an individual varies substantially over time” [43]. This perspective, which is essentially contrary to the traditional concepts in gut microbial ecology proposed by Savage 35 years ago [10], would have major conceptual implications on how we view the gut microbial ecosystem, as it proposes an important role of neutral processes in shaping the bacterial communities [44]. If correct, it would have repercussions that go beyond ecology and would impact how we conceive host-microbiota symbiosis and the microbiome’s role in health and disease. If host-associated microbiomes were mainly governed by neutral processes, it would be extremely difficult for the host to select and maintain beneficial symbionts over ecological and evolutionary time frames. The development of a beneficial relationship would be extremely unlikely, as mutualism is favored by the selection of true mutualists and their stable maintenance over evolutionary time [45]. Accordingly, a recent modeling approach predicted that in a neutral host without control over the bacterial symbionts, mutualism would be intrinsically fragile [46].

The temporal analysis of the fecal microbiota of three humans that we report here is more supportive of the traditional perspective on gut microbial ecology [10], as we were able to detect a stable temporal core comprising around 40 OTUs per human individual over a one-year period. In accordance to the findings by Caporaso et al. [28], this temporal core represents a very small fraction of the total OTUs. However, it is important to emphasize that it constitutes, quantitatively, the majority (>75%) of the microbial community. Therefore, our findings indicate that the human fecal microbiome is dominated by species that form stable populations over longer periods of time. This is consistent with the continuous detection of strains in human fecal samples [47], [48], [49], [50] and the stability of the microbiota described with alternative fingerprinting techniques [20], [21], [22], [23], [24], [29]. In addition, it appears that a stable microbiota is a general feature in mammals, as wild chimpanzees and lab mice also harbor stable microbial communities in their gut [51], [52].

Although this study revealed a temporal core, it is likely that the sequencing techniques used underestimate the relative proportion of this core. First, amplicon sequencing in combination with the bioinformatic approaches substantially overestimates diversity due to sequencing artifacts and limitations in data processing [42]. Most importantly, all current time- and processing-efficient cluster algorithms, including the one used in this study, lead to an over-splitting of OTUs (often 10–100 fold) [41]. This generates an artificial increase in α-diversity, and when different samples are compared, β-diversity, which was clearly demonstrated in our experiments with triple-associated mice (Figure S9). That methodological artifacts cause an artificial increase of OTUs and a concomitant reduction in the apparent fraction that are shared has been recognized by other researcher [42], but this notion has not been considered in studies that assessed inter- and intrapersonal microbiome variation [25], [28], and it is likely that these parameters have been over-estimated. Second, it has been shown that the size of the core microbiota is dependent on the depth of the analysis, and it is likely that the core, both within and between individuals, has been underestimated due to undersampling [53]. Accordingly, most of the dominant species detected in our study were persistent, while taxa present in lower abundance were generally detected in a smaller number of samples and were, therefore, not considered to be core members. However, given that most of these species were identified throughout the study period, it is likely that they represent stable members of the community occasionally falling below the detection threshold due to normal temporal fluctuations. However, despite the limitations of the sequencing approaches used in this study, a stable temporal core that dominated the fecal microbiome in humans was detected. In addition, the findings obtained in our mouse experiment indicate that a quantitative approach that focuses on abundance of OTUs can provide a more accurate representation of bacterial populations, and thus allows a better interpretation of community characteristics.

The temporal characteristics of the human fecal microbiota revealed during this study support the concept of an ecosystem that operates, to a large degree, in a state of homeostasis and shows resilience to perturbations caused by diet and lifestyle changes. Perturbations appear to mainly alter the relative proportions of the individual’s bacterial populations but do not cause extensive changes in its membership, as suggested by Rajilic-Stojanovic and co-workers [29]. Therefore, the gut microbiota appears to function as an ecological unit whose composition and local diversity is largely determined by niche-driven processes, a view that is also supported by theoretical model calculations [54], [55]. The significance of niches provides an explanation for ecosystem characteristics such as resilience and colonization resistance. Diet can alter the niche landscape through the provision of novel nutrients, leading to fluctuations in microbiome structure that are however reversible upon cessation of the dietary stimulus [30], . The use of antibiotics and invasions by better adapted microbes have the potential to remove members from the community, whose loss might alter the niche environment and may lead to more global and permanent changes [56]. The seemingly conflicting characteristics of the human gut microbiota, intra-individual stability despite substantial inter-individual diversity (even in mono-zygotic twins), can not only be explained by differences in host genotype, diet, and health, but also by a historical perspective of community assembly [14]. This view emphasizes the importance of historical patterns of dispersal and acquisition for the composition of the emerging community. These patterns are inherently stochastic, influencing colonization order and adaptive processes during assembly, thereby impacting the physicochemical properties of the niche-environment and the outcome of the assembly process. Inter-individual diversity could therefore be due to current and past personalized environmental differences (diet, antibiotics, exposure, host physiology, genetics and immunity, age, and metabolic state).

However, although our study confirmed that the gut microbiota is individualized, it also revealed a substantial inter-individual overlap, in terms of total sequences, between the microbiomes of the three subjects. The 16 stable OTUs present in the three individuals comprised, quantitatively, almost 50% of the sequences obtained from the individual subjects. Importantly, most of the stable species detected in our study in residents of the USA were also identified as core members in individuals residing in Europe [15], [50], and 27 of the species detected recently by Schloissnig and co-workers as dominant member of the human microbiome were also among the stable core in our study. Our findings suggested that the majority of the human fecal microbiota is composed of only around 69 species (Table 1), which is in accordance to recent findings obtained with whole metagenome sequencing that revealed that the gut microbiome of 207 individuals is composed of 66 bacterial species that account for 99% of the mapped reads [50]. These findings support the idea of a dominant core within human subjects [16], and these species could be considered autochthonous members of the human microbiota. In light of the novel sequence data, the requirements for autochthony proposed by Savage are probably too strict in that they call for members to be dominant throughout the entire life span of a host and present in all individuals of the host species [10]. It is unlikely that many lineages fulfill these requirements (also due to the impact of environment, diet, age, and health status on gut microbiota composition). However, the available data still support the concept of a human gut microbiota that is dominated by species that occupy long-term niches and are likely to share an evolutionary relationship with humans. Although the relative proportions of these lineages fluctuate and are susceptible to environmental cues and host physiology, they are still inherently human, are likely to play an important ecological role, and potentially support human health.

It is increasingly recognized that it will require the application of ecological theory to refine our understanding of the human microbiome, explain and predict community characteristics, and to inform strategies to successfully reshape the microbiota [6], [7], [8]. This study allowed inferences about the ecological principles that govern the human gut microbiota as it revealed that the human fecal microbiota is dominated by species that are temporarily stable and that overlap in individuals from the US and Europe. The temporal and spatial variation and dynamic nature of the gut microbiota revealed by next-generation sequencing has made some scientists focus on the plasticity of the gut microbiota and the lack of core species [6], [28], [43]. However, our evaluation of OTU-based sequencing approaches in gnotobiotic mice strongly suggested that much of this variation and the small apparent fraction of core species might be caused by methodological artifacts. We argue that the data obtained in this study, and most of the data that is now available (including metagenomic datasets [50]), is in favor of a stable human microbiota whose dominant members are, although in varying proportions, shared among a majority of humans in the western world. This view stresses the importance of niche-driven processes in shaping diversity and host control over the community [57], which would allow for a targeted selection of beneficial microbial communities during an individual’s life-span and over evolutionary times. Clearly, the development of ecological concepts that apply to the gut microbiota will require improved approaches for the characterization of microbial ecosystems that accurately determine diversity, and their careful interpretation, and efforts remain necessary to improve current tools.

## Materials and Methods

The human trials that are part of this study were approved by the Institutional Review Board of the University of Nebraska (IRB Approval Numbers: 2008038840EP and 2009019551EP), and written informed consent has been obtained from all subjects.

Triple-associated B6 mice were maintained at the University of Nebraska Gnotobiotic Mouse Facility and all experiments were performed with approval of the Institutional Animal Care and Use Committee (Project ID 731).

### Study Subjects and Experimental Design

Fecal samples of three individuals who had participated in two dietary trials conducted by our group [30], [31], [32] were included in this study. At the moment of the first sample collection subject 1 (female) was 25 years old, subject 2 (male) was 26 years old, and subject 3 (male) 23 years old. A total of 33 samples for subjects 1 and 2, and 32 samples for subject 3 were obtained throughout a 56-week time period. The time schedule of sample collection is shown in Figure 1. The first study was a randomized, double-blind, placebo-controlled, cross-over trial that lasted 17 weeks, in which crackers containing resistant starches types 2 or 4 (RS2 and RS4, respectively), or native starch, were consumed by the subjects. Weekly samples across the 17-week period were collected, including 2 samples at baseline, 3 samples per treatment (9 total) and 6 samples corresponding to 2-week washout periods between treatments and at the end of the final treatment [31]. 17 samples were thus collected for subjects 1 and 2, and 16 samples for subject 3 (one sample during a wash-out period was not collected). After a 24-week interval without sampling, a second trial was conducted in which weekly fecal samples were obtained throughout 16 weeks. The three subjects underwent 3-week treatments of increasing doses of galactooligosaccharides (GOS) (0 g, 2.5 g, 5 g, and 10 g), with additional 2-week baseline and washout sampling points at the beginning and end of the trial respectively [32].

The three subjects participating in these studies had no history of chronic gastrointestinal diseases, abnormal gastrointestinal symptoms, and did not take antibiotics 3 months prior to the beginning of each sampling period, or throughout the two sampling periods. Subject 1 received a 1-week antibiotic (clarithromycin) treatment during the 24-week period in which no sampling took place (week 19, corresponding to the 2^nd^ week of the non-sampling period).

### Characterization of the Fecal Microbiota by 454 Pyrosequencing

Both studies used the same protocols for sample collection, DNA extraction, and fecal microbiota characterization by pyrosequencing [31]. Briefly, the fecal bacterial community was characterized by massive parallel sequencing with the Roche Genome Sequencer GS-FLX using the Titanium platform (454 Life Sciences). The V1-V3 region of the 16S rRNA gene was initially amplified using a mixture (4∶1) of the forward primers B-8FM (5′- *CCTATCCCCTGTGTGCCTTGGCAGTCTCAG*AGAGTTTGATCMTGGCTCAG–3′) and B-8FMBifido (5′-*CCTATCCCCTGTGTGCCTTGGCAGTCTCAGAGGGTTCGATTCTGGCTCAG–3′* ), and the primer A-518R as the reverse primer (5′- *CCATCTCATCCCTGCGTGTCTCCGACTCAG*BBBBBBBBATTACCGCGGCTGCTGG–3′) (Martínez et al., 2010). The Titanium adaptor sequences (A and B) are shown in italics, and a unique 8-base barcode (BBBBBBBB) in the reverse primer was used to tag individual samples within the same run. Sequencing was performed following the manufacturer’s protocol.

Quality filtering and demultiplexing of the resulting sequence set was performed with the QIIME pipeline [58]. Sequences that were shorter than 300 or longer than 520 bases, contained one or more ambiguous nucleotides, had at least one mismatch to the primer or barcode, showed an average quality score below 25, or contained homopolymer runs over 6 bases, were removed. Chimeras were identified with the Blast Fragments Algorithm implemented in QIIME and removed. A total of 303509 sequences were obtained after quality controls and used for further analyses (108090, 107505, and 87914 for Subjects 1, 2 and 3, respectively). The sequences used for analysis can be found in the MG-RAST database, with the following accession numbers: 4521358.3, 4521394.3, 4521382.3, 4521416.3, 4521406.3, 4521437.3, 4521428.3, 4521415.3, 4521404.3, 4521432.3, 4521423.3, 4521414.3, 4521403.3, 4521395.3, 4521383.3, 4521373.3, 4521363.3, 4521393.3, 4521381.3, 4521356.3, 4521368.3, 4521377.3, 4521388.3, 4521398.3, 4521409.3, 4521419.3, 4521429.3, 4521400.3, 4521410.3, 4521424.3, 4521434.3, 4521402.3, 4521413.3, 4521418.3, 4521425.3, 4521436.3, 4521361.3, 4521370.3, 4521379.3, 4521389.3, 4521362.3, 4521372.3, 4521355.3, 4521366.3, 4521374.3, 4521385.3, 4521401.3, 4521412.3, 4521420.3, 4521430.3, 4521397.3, 4521408.3, 4521435.3, 4521427.3, 4521417.3, 4521407.3, 4521392.3, 4521378.3, 4521371.3, 4521359.3, 4521396.3, 4521384.3, 4521365.3, 4521353.3, 4521387.3, 4521375.3, 4521426.3, 4521391.3, 4521405.3, 4521422.3, 4521433.3, 4521411.3, 4521399.3, 4521431.3, 4521421.3, 4521364.3, 4521354.3, 4521386.3, 4521376.3, 4521367.3, 4521357.3, 4521390.3, 4521380.3, 4521369.3, 4521360.3.

Taxonomic assignments of sequences down to the genus level were made using the Classifier tool from the Ribosomal Database Project (RDP) [59]. Sequences were also designated to Taxonomic Operational Units (OTUs) with 97% similarity cutoff, using the Mothur v. 1.26.0 sequence analysis pipeline [60]. Clustering was performed with sequences from all three subjects in one single alignment. Very low abundance OTUs (5 sequences or less per subject) were removed from further analyses. The abundances of the phylotypes were computed as percent proportions based on the total number of sequences in each sample. OTU picking was also performed in the QIIME pipeline with the uclust algorithm (and default parameters), however, this procedure determined around double the amount of OTUs with QIIME (977+/−238 OTUs) when compared to MOTHUR (411+/−119 OTUs). Given that OTU picking algorithms have been shown to over-split OTUs [41], we used the amount of OTUs obtained with the two methods as a criterion to select the methodology and used the approach that resulted in the lowest amount of OTUs (MOTHUR). However, a visual inspection of the OTUs that represented the same phylotype showed that they followed the same temporal dynamics independent on the method used to generate the clustering (data not shown).

### Identification of the Stable Core Microbiota

OTUs detected in at least 80% of the samples within a subject were considered persistent members of the gut microbiota for that individual. Representative sequences of core OTUs were taxonomically classified at the phylum level with the Classifier tool. Next, pairwise alignments were performed for sequences within each phylum with ClustalW and default parameters in MEGA 5.05 [61]. Distance matrices were further constructed based on these alignments, and sequences with >97% similarity were combined within one OTU.

### Triple-associated Gnotobiotic Mice

We developed gnotobiotic mice from germ-free mice housed in the Gnotobiotic Mouse Facility at the University of Nebraska. *E. coli* MG1655 was streaked on MacConkey (BD) agar and grown aerobically overnight at 37°C. *Bifidobacterium adolescentis* BD1 and *Bacteroides thetaiotaomicron* VPI-5482 (ATCC-29148) were grown anaerobically on Rogosa SL (BD) (96 hours) and Bile Esculin agar (BD) (48 hours), respectively, at 37°C. We picked colonies from *E. coli*, *B. adolescentis*, and *B. thetaiotaomicron* and grew them in LB (BD) media, MRS (BD) with 5% L-cysteine, and TYG medium, respectively. *E. coli* was grown aerobically overnight (14 hours) at 37°C, while the other two cultures were grown anaerobically for 48 hours at 37°C. Cultures were washed once with PBS and mixed in volumetrically equal proportions immediately before inoculating germ-free C57BL/6 mice by allowing mice to drink the bacterial mixture and pouring it on their fur. Colonized mice were maintained and bred under gnotobiotic conditions.

The presence of the three bacterial species was confirmed by Denaturing Gradient Gel Electrophoresis (DGGE) (data not shown) and selective culture. DGGE also confirmed the absence of other bacterial species from the community. Selective culture was performed from fecal samples on Rogosa SL for *B. adolescentis*, Brain Heart Infusion (BD) with 10% sheep blood and 0.2 mg/mL gentamicin for *B. thetaiotaomicron*, and MacConkey for *E. coli*. Rogosa SL and Brain Heart Infusion plates incubated anaerobically at 37°C for 96 hours and 48 hours, respectively, before enumeration. MacConkey agar plates incubated aerobically at 37°C for 24 hours before enumeration. DNA from 1–3 fecal pellets per individual mouse was extracted following a conventional phenol-chloroform protocol with enzymatic and mechanic cell lysis [62]. Sequencing of the bacterial community, and quality processing of the sequences was performed as described above, using 1,000 randomly selected sequences per animal. Quality-controlled sequences were taxonomically characterized with the Classifier tool and OTU picking was done with MOTHUR and QIIME, as described above. QIIME resulted in less total OTUs then MOTHUR (185 versus 237), but produced more average OTUs per animal (51 versus 42). As MOTHUR was used to analyze the human data, we used the OTUs obtained with this software for the diversity analysis.

## Supporting Information

Figure S1 **Principal-coordinates plots of the beta-diversity measurements based on unweighted UniFrac distances among samples.** Samples were color-coded by subject (A) and by subject and trial period (B).
(PDF)

Figure S2 **Presence-absence patterns of dominant bacterial taxa in the fecal samples of human subjects over the entire study period.** Sequences were taxonomically classified (Classifier, RDP) and the presence (red) and absence (white) patterns of the most dominant bacterial taxa are presented for each sample at the phylum, order, family and genus levels. Samples are grouped by subject and presented in chronological order.
(PDF)

Figure S3 **Resilience of core members to dietary perturbations.** Abundance of *Bifidobacterium adolescentis* in fecal samples of (A) subject 1 (showing an increase in abundance due to the intake of both resistant starches and GOS) and (B) subject 2 (showing increase only with resistant starches) and (C) *Parabacteroides distasonis*, which was only significantly increased in subject 1 during consumption of resistant starch 4.
(PDF)

Figure S4 **Temporal dynamics of core taxa within the Actinobacteria phylum.** Abundances of the phylotypes identified as persistent (present in >80% of the samples) within subjects are presented in their phylogenetic context. A representative sequence of each OTU and the closest related type-strains were used to build trees with the neighbor-joining algorithm (1000 bootstrap replicates).
(PDF)

Figure S5 **Temporal dynamics of core taxa within the Bacteroidetes phylum.** Abundances of the phylotypes identified as persistent (present in >80% of the samples) within subjects are presented in their phylogenetic context.
(PDF)

Figure S6 **Temporal dynamics of core taxa within the Firmicutes phylum (Clostridia cluster XIV).** Abundances of the phylotypes identified as persistent (present in >80% of the samples) within subjects are presented in their phylogenetic context.
(PDF)

Figure S7 **Temporal dynamics of core taxa within the Firmicutes phylum (Clostridia clusters non-XIV).** Abundances of the phylotypes identified as persistent (present in >80% of the samples) within subjects are presented in their phylogenetic context. Phylogenetic trees were constructed as described in Suppl. Fig. 4.
(PDF)

Figure S8 **Invasion and extinction events within the fecal bacterial community.** The abundance of OTUs that displayed temporal dynamics that suggested events of invasion or extinction are shown. See text for details.
(PDF)

Figure S9 **Characterization of the fecal microbiota of triple-associated gnotobiotic mice.** Genus level classification (Classifer, RDP) of sequences obtained for each fecal sample of mice colonized by with *Bifidobacterium adolescentis* BD-1, *Escherichia coli* MG1655, and *Bacteroides thetaiotaomicron* VPI-5482 (A). Accumulated fraction of OTUs of total OTUs (blue line) and total sequences (red line) shared across all nine samples (B). The expected fraction and abundance represented by the broken black graph.
(PDF)
