# Supplementary figures and images for: Long-Term Temporal Analysis of the Human Fecal Microbiota Revealed a Stable Core of Dominant Bacterial Species
Source: PLoS One. 2013 Jul 16;8(7):e69621. doi: 10.1371/journal.pone.0069621 (PMC3712949; doi:10.1371/journal.pone.0069621)

A

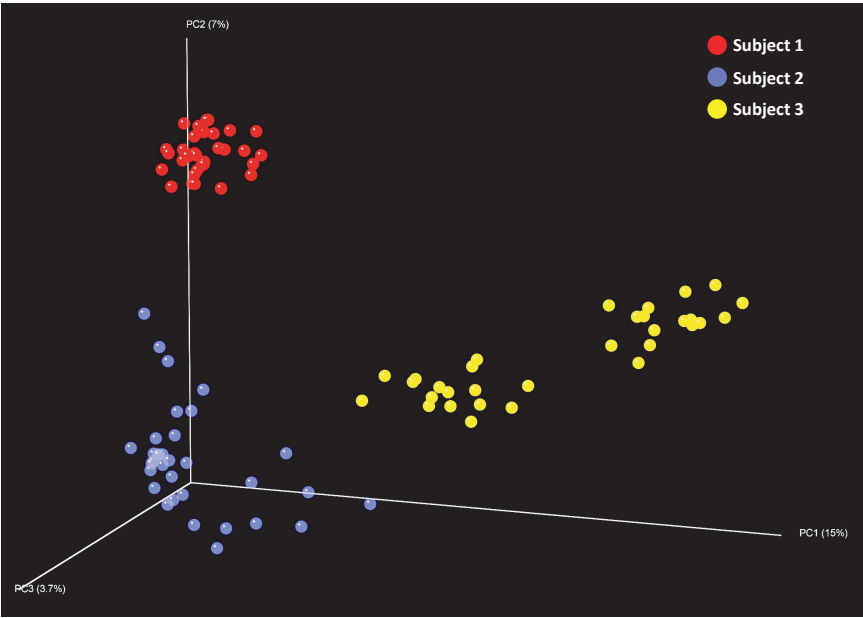

B

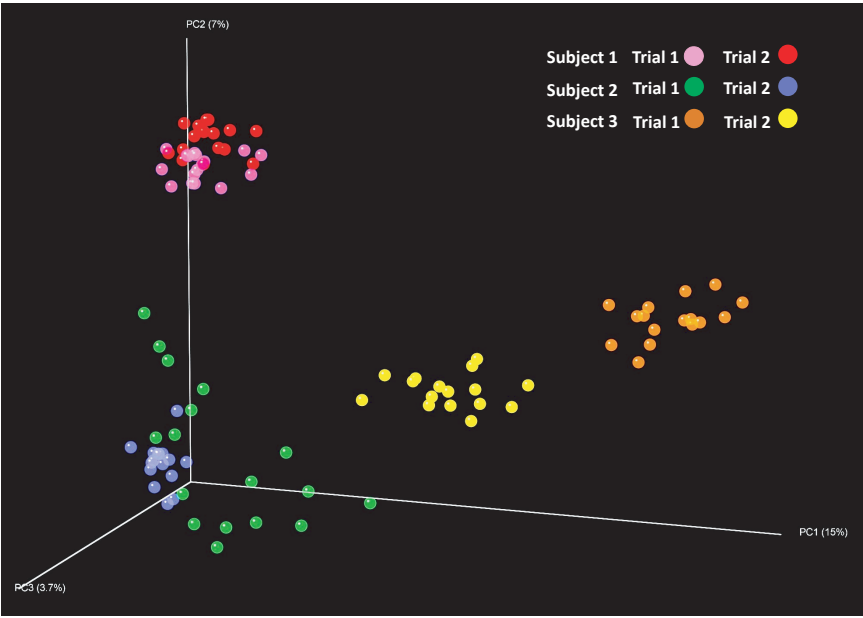

Supplement: Figure S1 — Principal-coordinates plots of the beta-diversity measurements based on unweighted UniFrac distances among samples. Samples were color-coded by subject (A) and by subject and trial period (B). (PDF) [file pone.0069621.s001.pdf]

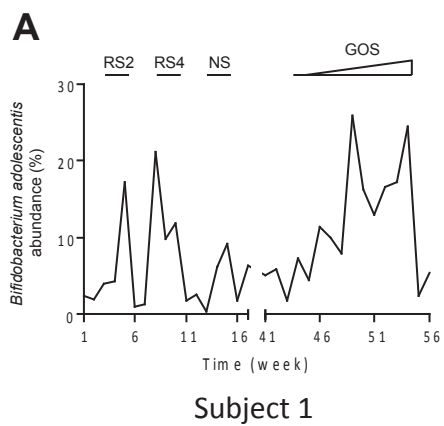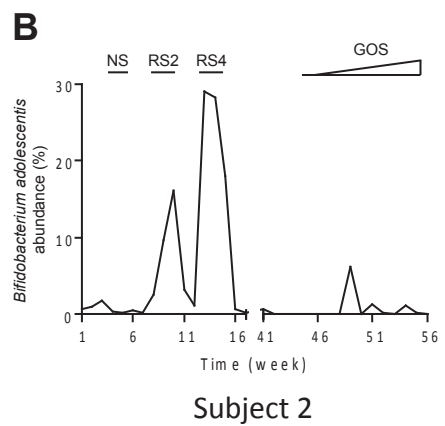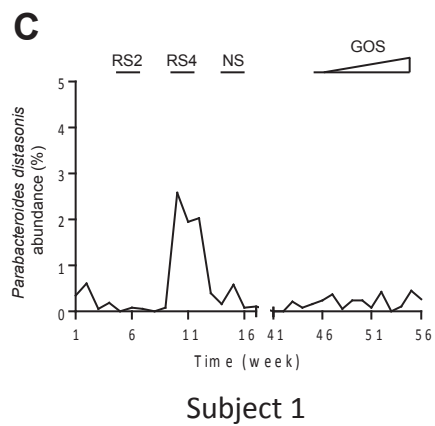

Supplement: Figure S3 — Resilience of core members to dietary perturbations. Abundance of Bifidobacterium adolescentis in fecal samples of (A) subject 1 (showing an increase in abundance due to the intake of both resistant starches and GOS) and (B) subject 2 (showing increase only with resistant starches) and (C) Parabacteroides distasonis, which was only significantly increased in subject 1 during consumption of resistant starch 4. (PDF) [file pone.0069621.s003.pdf]

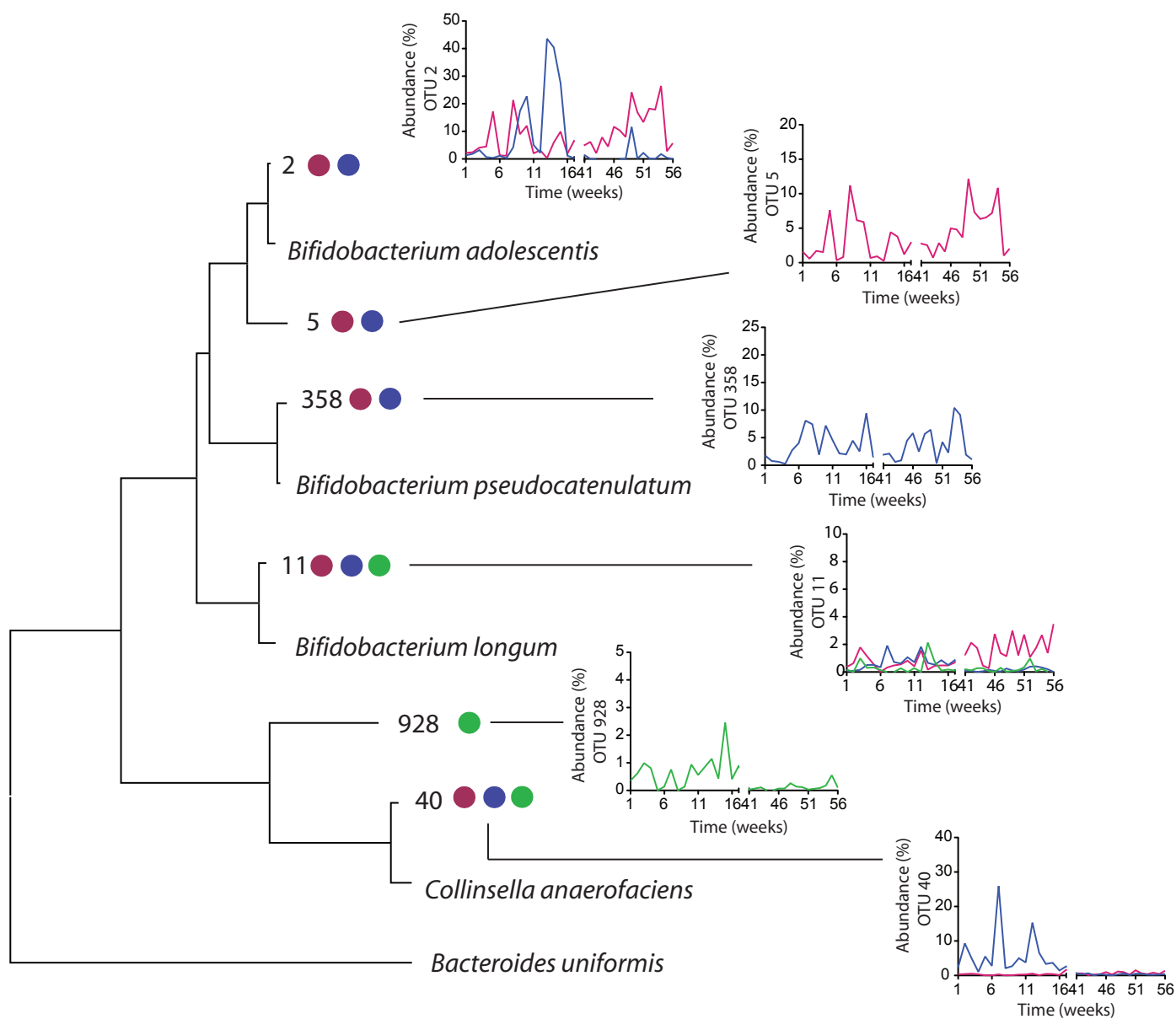

0.05

—●— S1  
—●— S2  
—●— S3

Supplement: Figure S4 — Temporal dynamics of core taxa within the Actinobacteria phylum. Abundances of the phylotypes identified as persistent (present in >80% of the samples) within subjects are presented in their phylogenetic context. A representative sequence of each OTU and the closest related type-strains were used to build trees with the neighbor-joining algorithm (1000 bootstrap replicates). (PDF) [file pone.0069621.s004.pdf]

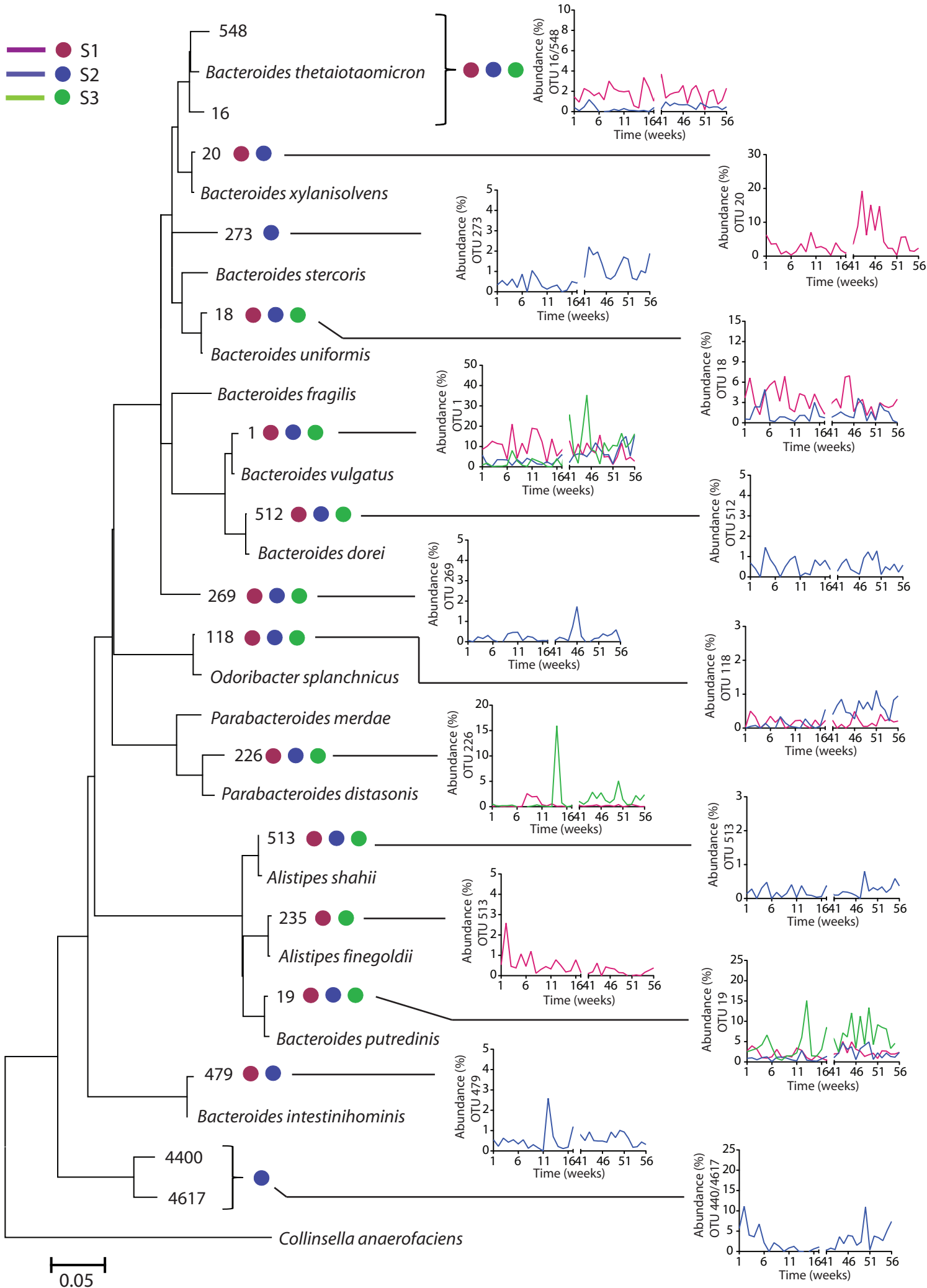

Supplement: Figure S5 — Temporal dynamics of core taxa within the Bacteroidetes phylum. Abundances of the phylotypes identified as persistent (present in >80% of the samples) within subjects are presented in their phylogenetic context. (PDF) [file pone.0069621.s005.pdf]

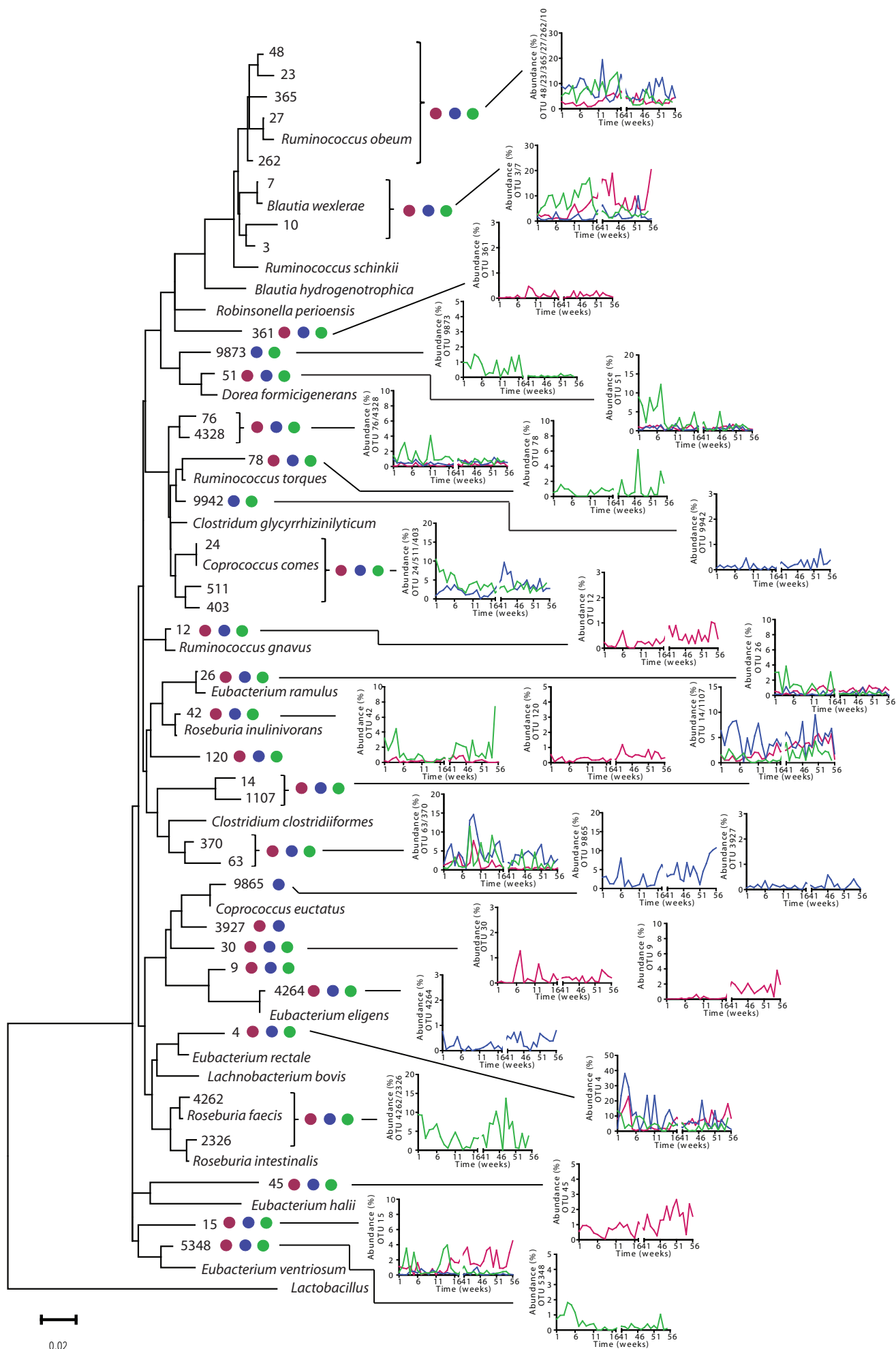

0.02

Supplement: Figure S6 — Temporal dynamics of core taxa within the Firmicutes phylum (Clostridia cluster XIV). Abundances of the phylotypes identified as persistent (present in >80% of the samples) within subjects are presented in their phylogenetic context. (PDF) [file pone.0069621.s006.pdf]

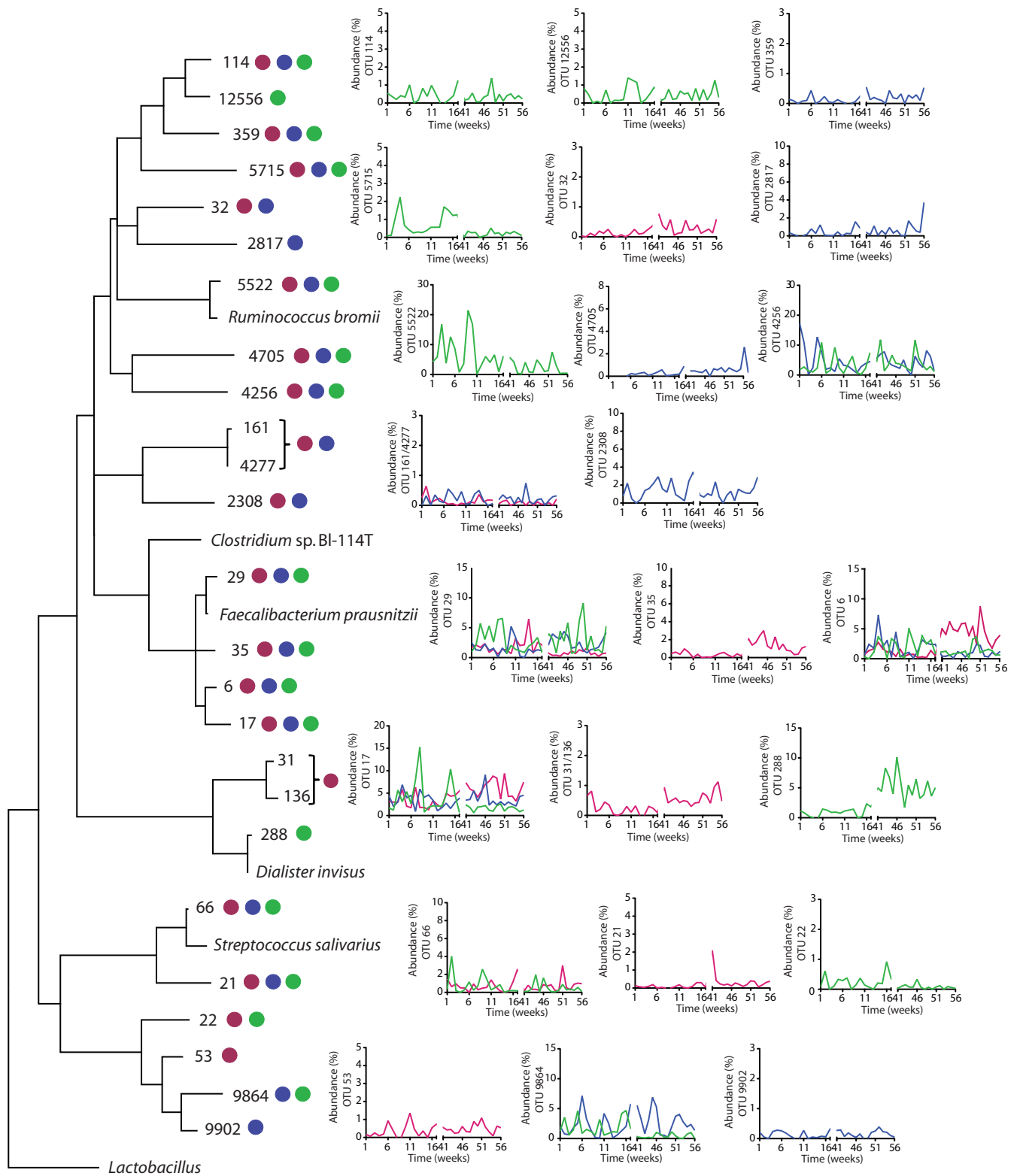

0.02

S1  
 S2  
 S3

Supplement: Figure S7 — Temporal dynamics of core taxa within the Firmicutes phylum (Clostridia clusters non-XIV). Abundances of the phylotypes identified as persistent (present in >80% of the samples) within subjects are presented in their phylogenetic context. Phylogenetic trees were constructed as described in Suppl. Fig. 4. (PDF) [file pone.0069621.s007.pdf]

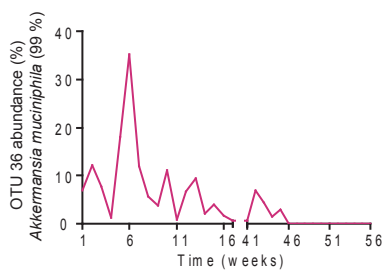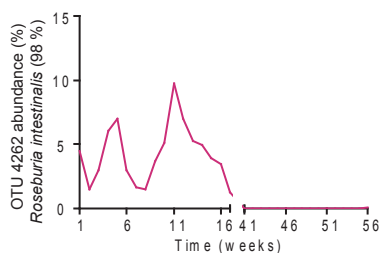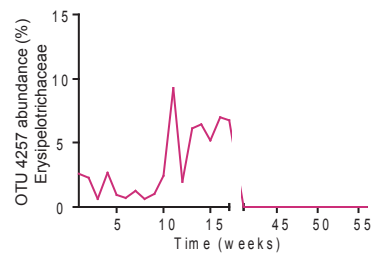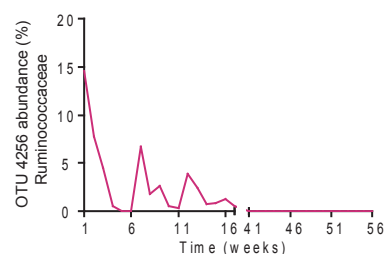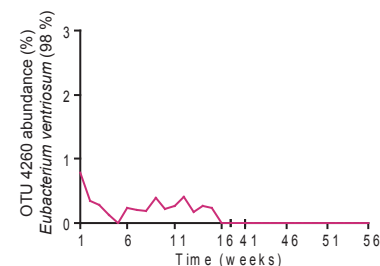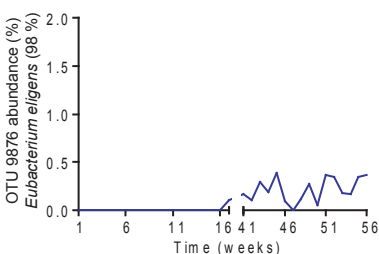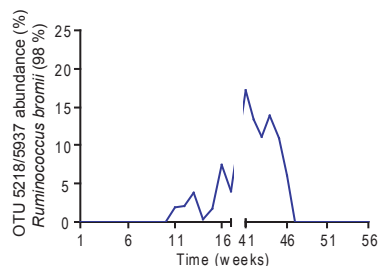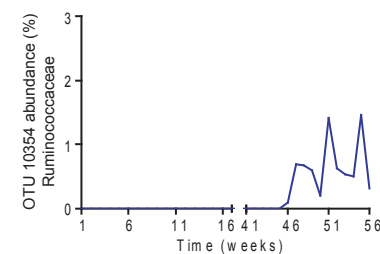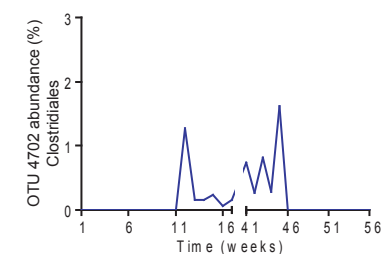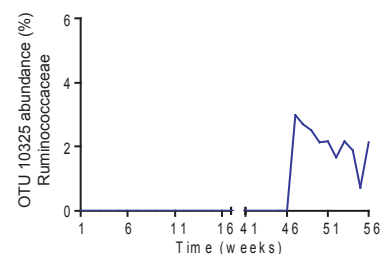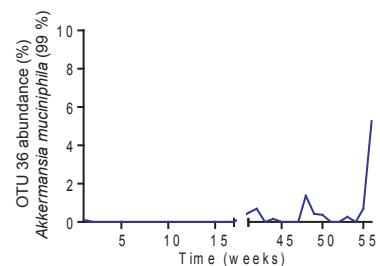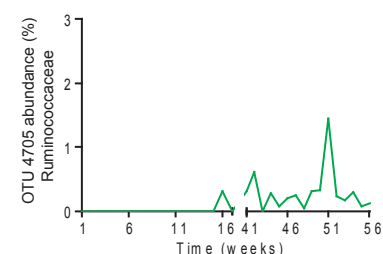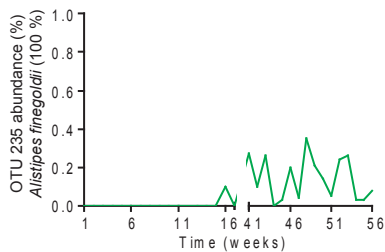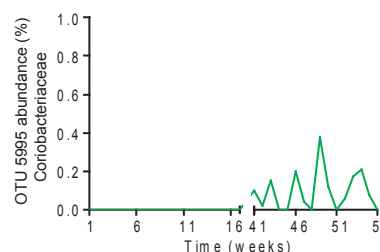

S1  
S2  
S3

Supplement: Figure S8 — Invasion and extinction events within the fecal bacterial community. The abundance of OTUs that displayed temporal dynamics that suggested events of invasion or extinction are shown. See text for details. (PDF) [file pone.0069621.s008.pdf]

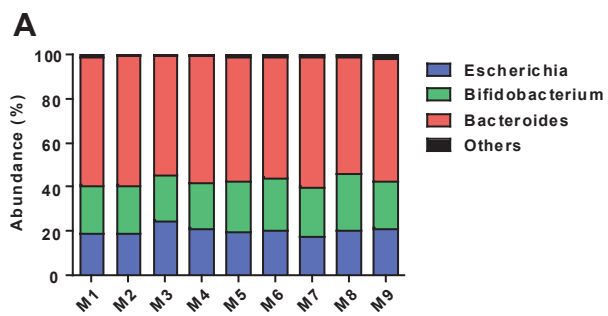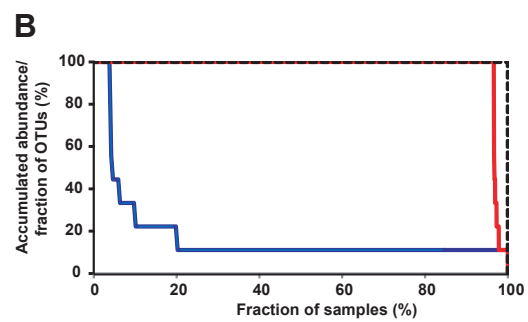

Supplement: Figure S9 — Characterization of the fecal microbiota of triple-associated gnotobiotic mice. Genus level classification (Classifer, RDP) of sequences obtained for each fecal sample of mice colonized by with Bifidobacterium adolescentis BD-1, Escherichia coli MG1655, and Bacteroides thetaiotaomicron VPI-5482 (A). Accumulated fraction of OTUs of total OTUs (blue line) and total sequences (red line) shared across all nine samples (B). The expected fraction and abundance represented by the broken black graph. (PDF) [file pone.0069621.s009.pdf]
