# Supplementary material for: Long-Term Temporal Analysis of the Human Fecal Microbiota Revealed a Stable Core of Dominant Bacterial Species
Source: PLoS One. 2013 Jul 16;8(7):e69621. doi: 10.1371/journal.pone.0069621 (PMC3712949; doi:10.1371/journal.pone.0069621)
Supplement: Figure S2 — Presence-absence patterns of dominant bacterial taxa in the fecal samples of human subjects over the entire study period. Sequences were taxonomically classified (Classifier, RDP) and the presence (red) and absence (white) patterns of the most dominant bacterial taxa are presented for each sample at the phylum, order, family and genus levels. Samples are grouped by subject and presented in chronological order. (PDF) [file pone.0069621.s002.pdf]

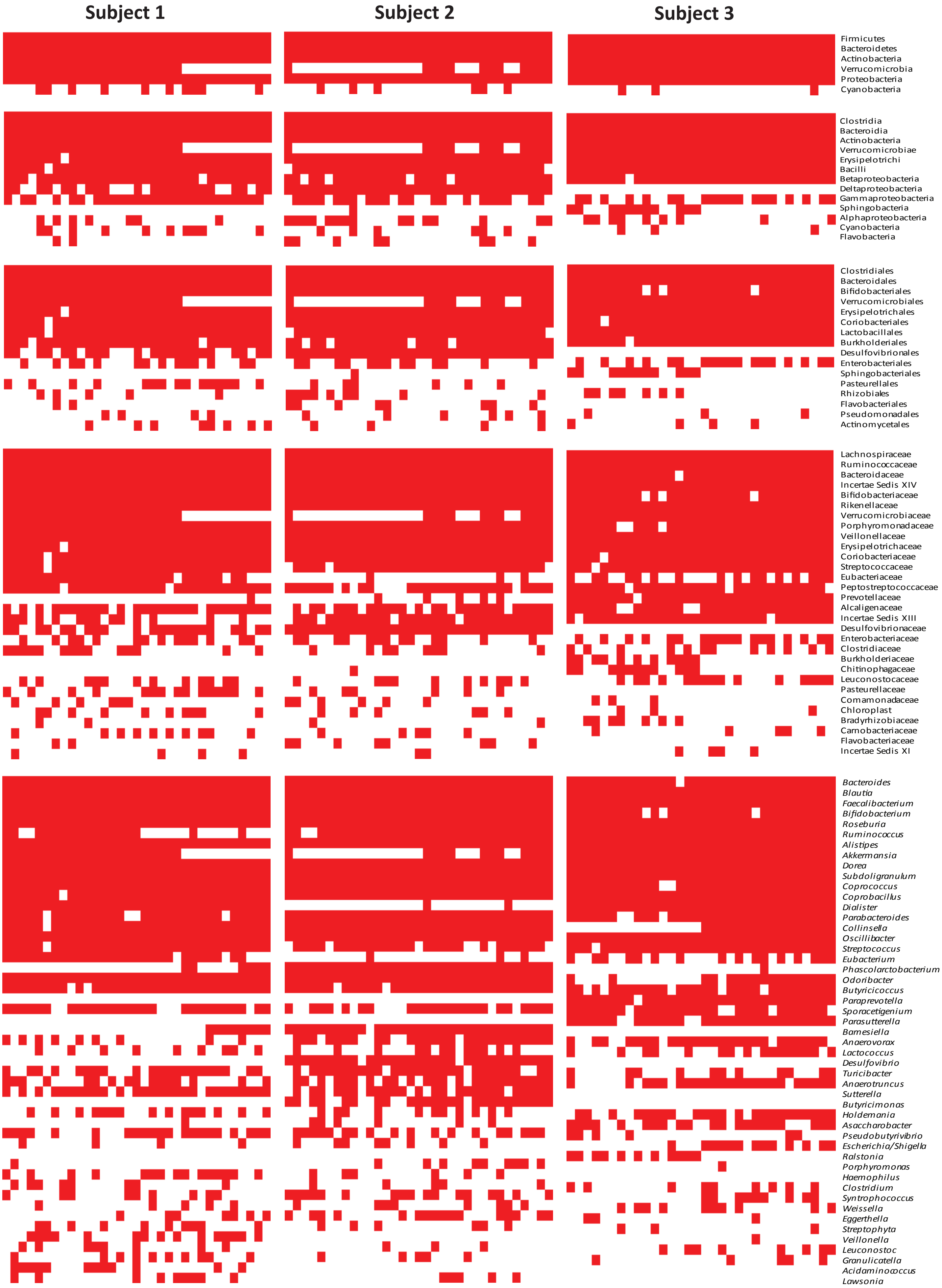
Subject 1
Subject 2
Subject 3
Firmicutes
Bacteroidetes
Actinobacteria
Verrucomicrobia
Proteobacteria
Cyanobacteria
Clostridia
Bacteroidia
Actinobacteria
Verrucomicrobiae
Erysipelotrichi
Bacilli
Betaproteobacteria
Deltaproteobacteria
Gammaproteobacteria
Sphingobacteria
Alphaproteobacteria
Cyanobacteria
Flavobacteria
Clostridiales
Bacteroidales
Bifidobacteriales
Verrucomicrobiales
Erysipelotrichales
Coriobacteriales
Lactobacillales
Burkholderiales
Desulfovibrionales
Enterobacteriales
Sphingobacteriales
Pasteurellales
Rhizobiales
Flavobacteriales
Pseudomonadales
Actinomycetales
Lachnospiraceae
Ruminococcaceae
Bacteroidaceae
Incertae Sedis XIV
Bifidobacteriaceae
Rikenellaceae
Verrucomicrobiaceae
Porphyromonadaceae
Veillonellaceae
Erysipelotrichaceae
Coriobacteriaceae
Streptococcaceae
Eubacteriaceae
Peptostreptococcaceae
Prevotellaceae
Alcaligenaceae
Incertae Sedis XIII
Desulfovibrionaceae
Enterobacteriaceae
Clostridiaceae
Burkholderiaceae
Chitinophagaceae
Leuconostocaceae
Pasteurellaceae
Comamonadaceae
Chloroplast
Bradyrhizobiaceae
Carnobacteriaceae
Flavobacteriaceae
Incertae Sedis XI
Bacteroides
Blautia
Faecalibacterium
Bifidobacterium
Roseburia
Ruminococcus
Alistipes
Akkermansia
Dorea
Subdoligranulum
Coprococcus
Coprobacillus
Dialister
Parabacteroides
Collinsella
Oscillibacter
Streptococcus
Eubacterium
Phascolarctobacterium
Odoribacter
Butyricicoccus
Paraprevotella
Sporacetigenium
Parasutterella
Barnesiella
Anaerovorax
Lactococcus
Desulfovibrio
Turicibacter
Anaerotruncus
Sutterella
Butyricimonas
Holdemania
Asaccharobacter
Pseudobutyrivibrio
Escherichia/Shigella
Ralstonia
Porphyromonas
Haemophilus
Clostridium
Syntrophococcus
Weissella
Eggerthella
Streptophyta
Veillonella
Leuconostoc
Granulicatella
Acidaminococcus
Lawsonia
